# Supplementary material for: Are carbon emissions trading and green financial instruments synergistic? -Comprehensive quantitative research based on content analysis
Source: PLoS One. 2024 Mar 7;19(3):e0298601. doi: 10.1371/journal.pone.0298601 (PMC10919601; doi:10.1371/journal.pone.0298601)
Supplement: S1 Data — (ZIP) [file pone.0298601.s001.zip › Data/地级市Z_F工作报告.zip!/╔╜╢1⁄2/╤╠╠¿╩╨/└┤╫╘╣1⁄2╓┌║┼í╛┬φ┐╦╩2╛▌═°í┐_2017.docx]

各位代表：

现在，我代表市人民政府向大会报告工作，请予审议，并请市政协委员和其他列席会议的同志提出意见。

一、第十六届政府工作回顾

2016年是第十六届政府的收官之年，在以习近平同志为核心的党中央坚强领导下，我们认真落实党中央国务院、省委省政府和市委各项决策部署，统筹推进“五位一体”总体布局，协调推进“四个全面”战略布局，强化“六抓六促”“六个持续”，积极应对经济下行压力，努力提高经济运行质量和效益，全市经济从三季度开始发生积极变化，全年生产总值增长8.1%，达到6926亿元，在全国城市中位次前移至第19位；规模以上工业主营业务收入增长7%，达到1.63万亿元，在全国城市中位次前移至第8位；一般公共预算收入增长8.6%，达到577.1亿元，占生产总值比重提高至8.33%；固定资产投资增长13.5%，达到5297.2亿元；社会消费品零售总额增长11.1%，达到2976.1亿元；进出口达到439.2亿美元，占全省近1/5；实际使用外资增长14.2%，达到21.6亿美元；城镇和农村居民人均可支配收入分别增长7.9%和7.6%，达到38744元和16721元，全市主要经济指标增速继续高于全国、全省和首批14个沿海开放城市平均水平，实现了“十三五”良好开局和本届政府顺利收官。

本届政府履职的五年，是极不平凡的五年，也是成绩卓著的五年。五年来，全市上下抢抓机遇、应对挑战，坚定信心、保持定力，牢牢把握稳中求进工作总基调，促进了经济中高速增长；深入贯彻新发展理念，促进了产业向中高端迈进；始终坚持以人民为中心的发展思想，促进了民生持续改善，进一步加快了经济转型升级和社会全面进步的发展进程，顺利完成了本届政府五年工作任务。

这是稳中向好的进程，我们始终坚持凝心聚力抓发展，经济总量和运行质量持续提升。全市生产总值年均增长9.2%，一般公共预算收入年均增长13.7%，规模以上工业主营业务收入年均增长9.4%，固定资产投资年均增长16.7%，社会消费品零售总额年均增长13%。产业结构不断优化，三次产业之比由7.4：57.7：34.9调整为6.7:50:43.3。深入实施“双百千”工程，培育起5个千亿级、17个百亿级产业集群，战略性新兴产业产值占到规模以上工业总产值的21.2%，是全省唯一连续六年工业技改投资过千亿的市、唯一连续11年完成节能减排目标的市、唯一国家消费品工业“三品”战略示范试点市，以全国地级市第1名的成绩获评工业稳增长和转型升级成效明显市。服务业占比提高8.4个百分点，新增各类金融机构49家，本外币存贷款余额分别达到7580亿元和4737亿元。13家农村信用社全部完成银行化改革。区域性基金管理中心建设方案获省批复，基金规模达到926亿元。入选首批国家全域旅游示范区创建单位，去年游客突破6500万人次。获评中国鲁菜之都称号。获批国家电子商务、跨境电子商务示范城市，去年电商交易额达到2775.8亿元。邮政业务收入年均增长30%，快递业务量增长11倍。粮食生产实现“十四连丰”，小麦高产创建屡次刷新全国纪录，烟台苹果连续8年蝉联中国果业第一品牌，农业机械化水平达到86%，成为全国首批海洋经济创新发展示范城市、全国海洋生态文明建设示范区、国家无规定动物疫病区，国家级农业龙头企业达到13家、居全国地级市首位。

这是厚植优势的进程，我们始终坚持振兴实体经济这个根基，构建起大中小企业协同发展格局。市场主体从28万户增加到71.6万户，主营业务收入过百亿元工业企业达到16家，高新技术企业由163家增加到370家。上市公司由30家增加到42家，“新三板”和区域股权交易市场挂牌企业分别达到73家和119家。万华集团MDI产能上升到全球首位。张裕葡萄酒蝉联国内市场占有率第1名。上汽通用东岳基地年产整车54万辆、动力总成134万台。富士康、乐金显示、浪潮乐金居全省进出口前3位。中集来福士深海钻井平台全球市场占有率上升到26%。南山集团入选世界著名品牌500强。丛林车体型材占中国高速列车市场份额扩大到50%。招金集团、中矿集团经济效益居全国黄金行业前列。玲珑轮胎建成运营全球认证的轮胎综合试验场。绿叶制药获评中国最具创新能力医药企业。东方海洋、瑞康医药等上市公司立足烟台、发挥资本优势进军新兴产业。京鲁船业成为国家远洋渔船建造与技术示范基地。万华、杰瑞、持久钟表入选国家制造业单项冠军企业。振华、万达广场、中粮大悦城等商贸综合体发展活跃。鲁花、龙大、喜旺、仙坛、双塔、民和牧业等企业有力带动了农民致富。广大企业家坚守主业、追求卓越，情系烟台、投资烟台、发展烟台，增创了我市产业发展的新优势。

这是动能转换的进程，我们始终坚持改革开放不动摇，发展活力动力不断增强。改革推动效应持续扩大，争取国家和省改革试点164项，推进国有企业、财税体制、农业农村、医药卫生及教育、科技、文化、价格等领域改革，实施税收“营改增”、不动产统一登记、公立医院等改革，开展公车改革。供给侧结构性改革取得阶段性成果，商品住宅去化周期持续降低，78条降成本措施初见成效。开放带动效应持续扩大，加快“五个国际化”进程，与“一带一路”沿线国家和地区全部建立经贸往来关系，累计实际使用外资87.6亿美元，引进世界500强企业24家、总数达99家。国际友城增加到24个。一批企业到境外开发资源、开拓市场，中匈宝思德经贸合作区上升为国家级境外经贸合作区。烟台开发区综合实力稳居全国219个国家级开发区第6位，烟台高新区获批山东半岛国家自主创新示范区，烟台保税港区同上海、天津、重庆3个直辖市同类园区并列进出口百亿美元方阵，招远开发区综合排名前移，创新设立中韩（烟台）产业园，昆嵛山保护区成为国家生态旅游示范区，东部新区拉开发展框架，4个省级开发区进入全省30强。创新驱动效应持续扩大，高新技术产业产值占比由38.7%提高到41.9%。引进3个国家级科研院所，新增6个国家级企业孵化器，获得19项国家科技奖励。评国家知识产权示范城市。

这是城乡统筹的进程，我们始终坚持强化基础设施建设，城镇化水平逐步提高。全市城镇化率由56.8%提高到62.1%，获批全国新型城镇化综合试点。城市总体规划获国务院批复。青烟威荣城际铁路建成投运，龙烟铁路主体完工，潍莱高铁开工建设，城市轨道交通环评报告通过国家审查。烟台蓬莱国际机场建成通航，去年航空旅客吞吐量突破500万人次，货邮吞吐量突破5万吨。烟台港西港区建成40万吨矿石码头、30万吨原油码头，至淄博输油管道全线贯通，全市港口货物吞吐量由2.4亿吨增加到3.5亿吨。建成烟海高速及3条疏港高速公路，启动建设蓬栖、龙青、文莱高速公路，新建改造干线公路1269公里、农村公路4120公里，建成胜利路南延、滨海西路、城铁南站南路等市区道路50多条。长岛南北长山大桥、丁字湾跨海大桥竣工通车。海阳核电一期基本完工，华电国际莱州电厂一期建成发电。胶东调水工程和南水北调配套工程建成供水，蓬莱至长岛实现跨海供水，完成11座大中型水库、301座小型水库除险加固。筹建保障房1.2万套，改造棚户区9.2万户。新建扩建垃圾处理项目6个、污水处理厂7座，改造地下管网1138公里，新增供热面积870万平方米、燃气用户37.9万户。15个镇成为全国重点镇，96个村居获评省级生态文明乡村。完成1.9万户农村危房和28万户农厕改造，村庄街道硬化率达到98%以上、自来水普及率达到96.1%。空气质量优良率达到85.8%，居全省第2位。重点河流监测断面水质达标率稳定在85%以上，门楼水库总氮指标平均浓度由2013年的每升14.36毫克下降到6.17毫克，其它指标总体达到Ⅱ类地表水标准。造林绿化110万亩，森林覆盖率达到40%，荣获全国绿化模范城市、国家森林城市称号。

这是共建共享的进程，我们始终坚持执政为民，社会民生事业取得新进展。民生支出占财政支出比重由71.4%提高到77.6%。城镇和农村居民人均可支配收入年均分别增长9.4%和10.7%。企业退休职工月人均养老金由1753元提高到2969元，失业金由每月600元提高到1000元，城乡低保县均标准由每人每年4500元和2079元提高到6708元和4188元。减少贫困人口13.4万人。新增城镇就业80.7万人、农村劳动力转移就业16.9万人。启动全民参保登记计划，整合城乡居民养老保险、医疗保险，建立职工和居民大病保险制度。成为首批国家健康城市和医养结合试点市，新增养老床位4.5万张。义务教育均衡发展走在全国前列，“全面改薄”和解决大班额改造校舍178.2万平方米。接受爱心捐款9.35亿元，救助困难群众117万人次。成功举办海阳亚沙会，烟台体育健儿在国内外重大赛事上屡创佳绩。编制实施文化体育设施规划和健康步道规划，胶东文化广场、全民健身广场等一批项目顺利建设，大中专院校体育设施向社会开放。成功创建国家历史文化名城、国家公共文化服务体系示范区，获评文化强省建设先进城市。蝉联全国文明城市“四连冠”，涌现出李登海、刁娜、刘盛兰、王福禄等一批时代楷模、道德模范、中国好人。信访、综治、维稳工作扎实有效，安全生产事故起数、死亡人数连续15年“双下降”，获批省食品安全示范城市，“法德共进”经验在全省推广，社会治安形势总体稳定，实现全国社会治安综合治理优秀城市“六连冠”。国防动员、民兵预备役、人民防空工作顺利开展，连续获评全国双拥模范城。援藏援疆和扶贫协作工作取得实效。人口计生工作保持全国先进水平，老龄、妇女、儿童、残疾人、红十字事业健康发展，统计、史志、档案、仲裁、机关事务、新闻出版、广播电视、民族宗教和气象、地震等工作取得新成绩。城市信用综合指数在全国地级市中位居第2位。

这是作风转变的进程，我们始终坚持从严治党、从严治政，政府自身建设得到加强。扎实开展党的群众路线教育实践活动、“三严三实”专题教育、“两学一做”学习教育，牢固树立政治意识、大局意识、核心意识、看齐意识，严格落实中央八项规定精神和省委实施办法、市委实施意见，驰而不息纠正“四风”。认真执行人大决议决定，主动向政协通报情况，办复市人大代表建议558件、政协提案2515件。加强法治政府建设，规范重大行政决策程序，建立法律顾问制度，推进行政复议规范化建设。深化“放管服”改革，11次取消下放市级行政权力事项345项，政务服务平台实现省市县乡四级互联互通。健全完善政务公开制度，获评网络履职绩效全国十佳城市。强化党风廉政建设和反腐败工作，加强行政监察和审计监督，政府队伍建设取得新成效。

各位代表，过去五年的成就是在经济下行压力加大的形势下取得的，困难中的转机来之不易，压力下的收获弥足珍贵。这是党中央国务院、省委省政府和市委正确领导的结果，是市人大、市政协及社会各界监督支持的结果，是全市上下努力拼搏的结果。在此，我代表市人民政府，向全市人民、各界人士，向驻烟人民解放军指战员和武警官兵，向中央省属驻烟单位，向老领导、老同志们，向所有关心支持烟台发展的海内外朋友们，表示衷心的感谢并致以崇高的敬意！

各位代表，在肯定成绩的同时，我们也清醒地看到，发展中还存在不少困难和问题。经济下行压力依然较大，大项目好项目不多，投资拉动不够强，稳定外贸压力较大，社会消费动力不足。经济结构还不够优化，服务业发展水平低，传统产业优化升级、新兴产业发展壮大的步伐不够快。县域经济发展不均衡，比较优势发挥不充分。财政刚性支出增多，收支平衡难度加大，支撑统筹发展的基础不牢固。企业整体创新能力不强，终端产品、名牌产品比重较低，产品附加值不高。中小微企业存在融资难、人才引进难的问题。城市交通拥堵问题日益显现，违法建设治理任务艰巨，城乡环境、生态保护以及住房、教育、医疗、养老、收入分配等方面与群众期盼还有差距。少数干部懒政怠政和不作为、慢作为的现象仍然存在，一些领域腐败问题时有发生。对这些问题，我们将坚持标本兼治，采取有力措施，认真加以解决。

二、第十七届政府任务目标

刚刚闭幕的市第十三次党代会确立了未来发展的总体思路和宏伟目标，描绘了今后五年的美好蓝图。我们要按照市党代会的总体部署，高举中国特色社会主义伟大旗帜，以马克思列宁主义、毛泽东思想、邓小平理论、“三个代表”重要思想和科学发展观为指导，在以习近平同志为核心的党中央领导下，全面贯彻党的十八大和十八届三中、四中、五中、六中全会精神，深入贯彻习近平总书记系列重要讲话和视察山东重要讲话、重要批示精神，统筹推进“五位一体”总体布局，协调推进“四个全面”战略布局，贯彻落实新发展理念，以提高质量和效益为中心，坚持稳中求进工作总基调，把握供给侧结构性改革这条主线，大力振兴实体经济，全面做好稳增长、促改革、调结构、惠民生、防风险各项工作，促进经济总量、运行质量和人均均量“量质齐升”，实现政府财政、企业利税和居民收入“财富齐增”，努力推动烟台率先发展、走在前列，奋力打造宜居宜业宜游的成功城市。

各位代表，市党代会确定的奋斗目标，是民心所盼、发展所向，新一届政府要紧紧围绕实现这个目标，聚心惠民、聚力兴企、聚焦强市，推动烟台走向人、产、城“三位一体”，生产、生活、生态“三生协调”，宜居、宜业、宜游“三宜融合”的发展道路，让经济更加繁荣、人民更加幸福、社会更加和谐、城乡更加秀美，率先全面建成小康社会。

未来五年，我们要“聚心惠民”，努力建设幸福和谐家园，切实增强全市人民的获得感、自豪感。认真落实共享发展理念，稳步提高民生支出占财政支出比重，持续提升社会保障水平，大力发展以扶老、助残、救孤、济困为重点的社会福利事业，建立健全科学规范的扶贫脱贫长效机制，确保托住底、让所有人基本生活无忧。保持居民收入增长与经济增长同步，提高基本公共服务均等化水平，做好群众普遍关心的教育、医疗、住房、治理交通拥堵等民生工作，居民人均可支配收入年均增长8%左右，努力增实惠、让更多人过得好。创新发展健康、养老、文化、体育、旅游“五大幸福产业”，城镇社区形成15分钟文化健身圈、医疗服务圈、便民生活圈，空气质量达到国家二级标准，重点河流达到水环境功能区要求，森林覆盖率达到42%以上，法治、平安、和谐烟台建设上升到新水平，让全市人民始终感到生活有新变化、新奔头。

未来五年，我们要“聚力兴企”，努力营造尊重支持创造创业、尊重支持企业发展的良好环境。坚持不懈大力发展实体经济，促进优势资源向潜力企业集中、向行业龙头聚集。着力扶持企业发展，降低企业成本，帮助企业争取重大专项和扶持政策，搭建一批技术创新平台、科技孵化平台、基金支撑平台、服务保障平台，对近几年新兴的市场主体和现有企业分类精心培育、精准扶持，促进“个转企、小升规、规改股、股上市”。努力优化发展环境，进一步转变政府职能，整合部门责权，对企业办理事项实行分类归口部门代办制度，强化政务服务、政策支持、人文关怀，把烟台打造成行政审批最少、服务效率最高、营商环境最好的城市之一，争创国家社会信用体系建设示范城市。积极构建“亲”“清”新型政商关系，做到亲不逾矩、清不远疏，完善政府联系服务企业制度，以实际行动关心企业、尊重企业家、关爱产业工人，加强产权保护，使企业家安心经营、放心投资。我们期盼烟台企业持续壮大，邀请烟台籍工商界人士回乡兴业，欢迎八方有志之士来烟台投资发展。未来五年，再培植一批销售额超百亿元的企业，再发展一批市值超百亿元的上市公司，再引进一批投资过百亿元的产业项目，再打造一批纳税额超10亿元的纳税大户，让各类企业在烟台的广阔天地蓬勃发展。

未来五年，我们要“聚焦强市”，努力打造国际制造名城，全面提升产业竞争力和城市影响力。积极探索新时期强市之路，发挥实体经济基础优势，加快由工业大市向先进制造业强市迈进，落实《烟台市贯彻＜中国制造2025＞打造烟台智造行动纲要》，改造提升五大传统产业，突破发展四大优势产业，培育壮大三大新兴产业，到2021年规模以上工业主营业务收入突破2.5万亿元，高新技术产业产值突破1万亿元。围绕实施“一带一路”战略，加快由首批沿海开放城市向“一带一路”战略支点城市迈进，深化与沿线国家和地区经贸合作，形成开放型经济新优势，五年实际使用外资120亿美元以上，到2021年进出口达到600亿美元以上。实施陆海联动，加快由蓝色经济大市向蓝色经济领军城市迈进，打造海工装备自主创新集成基地、海洋生物产业基地和现代渔业基地。依托农产品资源和品牌优势，加快由食品大市向国内外食品名城迈进，建成农产品质量安全市、国家食品安全城市。挖掘独特旅游资源，加快由国内重要的旅游城市向国际知名的滨海旅游休闲度假城市迈进，创建国家全域旅游示范区，到2021年旅游总人数达到1亿人次。突破瓶颈制约，加快由国内重要港口城市向国际性航运物流中心迈进，推进“两港西迁”，形成“三港一站”优势，拓展北部沿海“一线七港”发展空间，统筹推进港口有机融合，全面提升海陆空联运功能。落实《山东半岛城市群发展规划》，加快由市区融合发展向中心城市群一体化发展迈进，实现联动发展、差异发展、共赢发展。加大城市宣传推介力度，讲好烟台故事，传播烟台声音，提升城市知名度。

聚心惠民、聚力兴企、聚焦强市，需要突破重点、带动全局。一是着力引进重大项目，坚定不移地把招商引资作为头号工程，全面提升环境、人才、产业聚集、配套服务综合竞争力，加大力度引进一批战略性支撑性重大产业项目。二是着力打造重大载体，实施功能区发展战略和新一轮县域经济提升计划，推动城市“科技东进、产业西进”，以东部各区为主体，打造高新技术产业引领区、高端服务业聚集区和蓝色经济示范区；以西部各区市为主体，打造先进制造业核心区、开放型经济龙头区和产城融合西部新城，把烟台开发区建设成为烟台现代化经济中心。三是着力突破重要产业，集中资源培育医药健康、精细化工、航空航天、核电装备4个千亿级产业集群，打造在国内外具有影响力的国际生命科学创新示范区、全球最具竞争优势的聚氨酯产业基地、国家通用航空产业发展示范区、中国核技术自主创新集成基地。四是着力实施重大工程，争取潍莱高铁尽快通车、环渤海高铁及早启动、城市轨道交通早日建设，规划建设塔山南路、塔山北路两条东西主干道，论证建设中心城市大外环高速公路，积极争取渤海海峡跨海通道项目，推进烟台港“一突堤”区片高标准开发以及烟台山、朝阳街、所城里保护性开发。五是着力抓好重大事项，深入谋划和推进事关烟台长远发展的重要工作，完善从蓬莱阁到昆嵛山滨海一带规划布局，加快智慧城市建设，论证核电热能和LNG冷能利用，争取设立国家现代农业产业园，促进烟台格罗宁根大学建成高水平研究型大学，筹建哈尔滨工程大学（烟台）研究生院，大力引进更多海内外工科院校、科研院所，支持驻烟高校“双一流”建设。六是着力争取重大政策，主动对接国家战略和政策导向，深入研究突破方向，千方百计争取政策、推进改革，抢占政策高地。国家级和省级园区都要强化体制机制优势，探索、复制创新政策，保持旺盛发展活力，继续在改革开放创新中走在前列。

各位代表，今后五年，我们要在实现“十三五”时期“一二三四五”总目标的基础上，全面完成市党代会确定的奋斗目标。到2021年，全市生产总值实现万亿元以上、年均增长8%左右，并力争夺取更好的发展成果。我们要在实现我们党第一个百年奋斗目标的征程上，率先全面建成小康社会；我们要在实现我们党第二个百年奋斗目标的征程中，书写好中华民族伟大复兴中国梦的烟台篇章。目标引领前进方向，目标凝聚奋斗力量。只要我们团结一致、坚定信心，迈好脚下的每一步，做实手中的每件事，就一定能够如期实现奋斗目标、创造更加美好未来!

三、2017年政府重点工作

今年是新一届政府开局之年，全市经济社会发展主要预期目标建议为：生产总值增长8%左右，一般公共预算收入增长8.5%左右，固定资产投资和社会消费品零售总额均增长11%左右，进出口保持稳定增长，实际使用外资增长3%左右，城镇居民人均可支配收入增长8%左右，农村居民人均可支配收入增长8%以上，完成省下达的节能减排降碳约束性指标。

（一）以改善供给为主攻方向，深入推进“三去一降一补”。扎实有效去产能，严格监管水泥、轮胎等产能过剩行业，淘汰落后产能。结合实际去库存，合理控制土地出让时序和规模，提高住房货币化安置比例，给予本地进城购房农民补贴优惠，使商品住宅去化周期逐步降到合理区间。积极稳妥去杠杆，促进企业盘活存量资产、实行资产证券化，支持市场化法治化债转股，开展政府债务置换。多措并举降成本，全面清理规范政府性基金，削减涉企行政事业性收费，减少政府定价的涉企经营性收费，降低制度性交易和用能、物流成本，落实小微企业享受减半征收所得税优惠政策。精准加力补短板，提升公共服务、基础设施、创新发展、资源环境等支撑能力，高标准高质量全面完成脱贫任务。

（二）围绕适度扩大总需求，抓好有效投资、挖掘消费潜力。推进总投资超过8000亿元的200个市级重点项目，完成年度投资1600亿元以上。引导民间资本投向实体经济和交通、水利、市政公用事业等基础设施及社会事业领域，建设市级中水回用工程等一批PPP项目。开通运营龙烟铁路，争取开工建设大莱龙铁路扩能改造工程，力争启动城市轨道交通建设。实现海阳核电1号机组并网发电。建成蓬栖高速公路，加快建设龙青、文莱高速公路，实施13个国省干线公路项目。开展“十大扩消费行动”，稳定大宗消费，扩大农村消费，发展新兴消费。鼓励开展商业模式创新，推动实体店销售和网购融合发展，建设2个县级电商服务中心、100个村级电商服务店，促进电商、快递进社区、进农村。

（三）推进农业供给侧结构性改革，创新发展绿色农业。实施果品、水产品、畜禽、蔬菜、茶叶等优势特色产业提质增效行动计划，拓展产业链价值链，推进一二三产业融合发展。引导农民根据市场需求发展生产，增加优质绿色农产品供给，发展地理标志保护产品。推进“粮安工程”和“海上粮仓”建设，发展高产示范田110万亩、海洋牧场示范区30万亩、高效节水灌溉面积17万亩、水肥一体化面积16万亩。培育优质种子种苗，加快苹果等农产品改良换代，开展畜禽养殖标准化示范创建活动。推进农业规模化、集约化、产业化经营，支持农业龙头企业做大做强，新发展农业合作社1000家、家庭农场500家，带动农民增收致富。实施农机创新示范工程，农业机械化水平达到90%以上。推进雨洪资源和水系连通工程，完成5座大中型水库、实施170座小型水库除险加固工程，协调组织调引客水工作，提高城乡供水保障能力。培育新型农民，支持农民工返乡创业，鼓励高校毕业生、退役军人、科技人员到农村施展才华。

（四）加快发展先进制造业，大力振兴实体经济。深入实施“双百千”工程和“五个一百”智能制造提升工程，推进“两化融合”，改造提升机械制造、电子信息、食品加工、黄金、现代化工等支柱产业，实施121个投资过亿元的技改项目。深耕医药健康产业，加快建设一批生物医药科技创新平台和产业园区，创办医药创新与发展国际峰会。深耕精细化工产业，推进万华工业园二期建设，加快化工企业“进区入园”步伐。深耕航空航天产业，着力建设航空材料产业园，培强做大通用航空、航空物流和航空旅游产业。深耕核电装备产业，扩大与核电企业合作，加快建设国家级核电研发中心和两个省级核电装备产业园。实施新产业倍增计划和创新示范工程，建设33个省级战略性新兴产业项目，发展信息经济、网络经济、分享经济，培育大数据、云服务等新产业，创新总集成总承包、个性化定制等新模式。建设国家电子商务示范城市，在工程机械、特色农产品海产品、化工建材、葡萄酒、金银饰品等领域打造一批线上产业集群。支持企业开展规范化公司制改制，推动更多企业上市和挂牌。创建全国质量强市示范城市，开展质量提升行动，弘扬企业家精神和“工匠精神”，支持企业参与国家标准和国际标准制定，推动内外销产品“同线同标同质”，创建更多自主品牌、区域品牌和驰著名商标、国际注册商标。

（五）促进三次产业融合，推动服务业提质增效。开展新一轮服务业综合改革试点，支持社会力量提供教育、养老、医疗等服务。精细化打造全域旅游示范区，做精做美中心城市“滨海一线”和“蓬长龙”重点旅游板块，推进75个在建旅游项目，开展“烟台人游烟台”活动，免费开放一批国有景区。推动地方金融组织提质增效，扩大新型农村合作金融试点范围，支持银行、保险等金融机构健康发展，增强支持实体经济能力。加快区域性基金管理中心建设，促进基金与产业融合发展。健全完善金融管理协作机制，有序化解处置风险，优化金融生态环境。促进商贸住餐、物流、会展、科技信息、社区服务、健康养老等行业繁荣发展。

（六）深化基础性关键性改革，进一步完善体制机制。实施国家、省试点政策，积极争创全国海洋经济发展示范区等新试点。深化国企国资改革，发展混合所有制经济，完善公司法人治理结构。落实财税体制改革，划分财政事权与支出责任。稳妥推进价格机制改革，构建烟台特色优势产品价格指数体系。稳步开展事业单位改革，推进公立医院、科研院所法人治理结构建设。开展综合行政执法体制改革。实施农村集体产权制度改革，推进农村土地“三权分置”，完成国有林场改革，深化供销社综合改革。

（七）加大对外开放力度，提高开放型经济水平。实施新一轮引资引技引智优惠政策，打造招商引资升级版。常态化、精准化开展“大走访”，瞄准世界500强和行业领军企业实施靶向招商，深化与央企、大型民企、科研院所对接合作，力争新引进世界500强投（增）资项目10个以上。推动外地企业把在烟分支机构转为法人企业，鼓励企业在烟设立总部，支持有实力的企业开展海外并购和境外经贸合作区建设。推动对外贸易转型升级，实施加工贸易提质增效、品牌商品出口促进计划，扩大机电、高新技术和品牌产品出口。加快省级服务贸易创新试点市建设，打造服务贸易公共服务平台。规划建设跨境电商综合园区，年内园区企业突破500家，建设一批出口产品公共“海外仓”。加快复制推广自贸区改革试点政策，推动外贸便利化改革和外汇便利化政策落地，全面推行一次性通关新模式，实现国际贸易“单一窗口”全覆盖。实施国际友城合作深耕计划，承办好第12届世界就业联盟东北亚区域会议等活动。

（八）大力推进科技创新，积极创建创新型城市。发挥山东半岛国家自主创新示范区优势，探索实施科技金融、成果转移转化等政策试点，建设提升丝绸之路高科技园区联盟、中俄和中格国际技术转移中心等创新平台。培育壮大创新型企业集群，新增高新技术企业60家以上、科技型中小企业140家以上。支持企业开展产学研合作，加强对企业人才和高层管理人员的培训，创建区域性创新中心。建设各类众创空间50万平方米，科技企业孵化器面积突破100万平方米。整合建设高精尖检测平台，更好地服务企业发展。加强知识产权保护，争取设立中国（烟台·食品）知识产权保护中心。实行“烟台优才卡”制度，创新人才政策，加快引进培育产业领军人才、科技创业人才和高技能人才。

（九）稳步实施城市有机更新，统筹推进城乡协调发展。高起点精准化规划提升城市品位，科学编制城市中长期规划和综合交通等专项规划。高标准精品化建设打造城市经典，实施219个市区城建重点项目，改造棚户区2.77万户、老旧小区6.2万户，搞好地下综合管廊和海绵城市建设，完成350公里供水供热管网、17平方公里雨污分流、3.6万户天然气改造任务，新增供热面积100万平方米、燃气用户2万户，建设一批居民区小型垃圾压缩站，逐步推进既有住宅加装电梯。高水平精细化管理保障城市运行，集中开展违法建设、铁路和高速公路沿线环境整治行动，着力改善市容市貌，提高公交分担率，加强交通管理，缓解拥堵问题。推进新型城镇化综合试点，深化户籍制度改革，加快“三个市民化”进程，城镇化率达到63%。加强乡镇政府服务能力建设，促进服务资源高效配置和有效辐射，抓好6个省级以上特色小镇创建工作。深入开展农村人居环境综合整治，治理乡村白色污染，改造村级公路800公里、县乡公路200公里，完成农村改厕16.2万户，解决695个村水质保障问题，实施200项库区扶持项目、7个省级美丽移民村项目，加强特色村庄建设和传统村落保护，建设既有现代文明、又具田园风光的美丽乡村。

（十）加强生态保护，持续改善环境质量。实施蓝天行动，深入开展燃煤、扬尘、机动车尾气等领域整治行动，推进煤炭清洁高效利用和散煤治理，完成28个燃煤机组超低排放改造，淘汰市区120台分散燃煤锅炉。实施清水行动，加快全国水生态文明城市建设，“一河一策”落实河长制。推进10大类23项市区饮用水源地保护工程，确保到2018年市区集中式饮用水水源地水质总体达到Ⅱ类地表水标准，总氮指标达到省内同类地区先进水平。实施26条重点河流控制单元达标工程，完成10条黑臭水体达标整治任务，改造升级9个污水处理厂，开展套子湾等17个海洋综合整治修复项目，基本消除劣Ⅴ类水体。实施绿地行动，开展森林县市区、森林镇村创建工作，加强自然保护区管理，绿化裸露土地，完成造林修复12.5万亩，保护恢复湿地6万亩，治理水土流失27万亩。开展土壤修复试点，防治农业面源污染，推进耕地质量提升保护工程。实施“工业绿动力”计划，培育一批绿色工业企业和园区，发展节能环保产业和循环经济。创建国家低碳城市，抓好中瑞低碳项目建设，推广装配式建筑和绿色建筑，扩大节能减排成果。倡导应用清洁能源，新增新能源公交车1100辆，通过国家绿色交通城市验收。严格环境执法和督查问责，对偷排、造假的，必须严厉打击；对执法不力、姑息纵容的，必须严肃追究；对环境质量恶化、应对不力的，必须严格问责。

（十一）织密扎牢社会保障网，兜住民生底线。建立困难群众基本生活保障协调机制。统筹推进高校毕业生和困难群体就业创业，新增就业13.3万人，城镇登记失业率控制在3.5%以内。继续提高退休人员基本养老金。完成全民参保登记，健全居民养老保险参保缴费激励机制，提高居民医保财政补助标准。深化医保支付方式改革，将18种靶向药和特效药纳入居民大病保险报销范围。实施民生综合保险工程，公共财政对城乡居民投保人身意外伤害险、对家庭投保财产损失险。整合农村五保供养和城市“三无”人员救助制度，实施城乡特困人员救助供养，发展慈善事业。保障老年人、妇女、儿童和残疾人权益，关爱保护进城务工人员子女、家庭困难青少年等弱势群体。

（十二）推动社会事业全面进步，促进公共资源公平共享。推进基础教育综合改革，实施职业教育产教融合工程，制定高等教育发展战略，建设省级德育课程一体化改革试验区。完成“全面改薄”和解决大班额任务，新建改建学校78所、校舍55万平方米。落实第二期学前教育三年行动计划，新建改建公办幼儿园72处。积极创建全国健康城市，开展全民健身、疾病防控等9项健康行动，大力发展体育产业。基本建成分级诊疗服务体系，健全院前急救医疗网络，完善家庭医生签约服务机制。提高公立医院基本公共医疗服务水平，依托重点公立医院建设一批特色专科医院，推进10个县市区妇幼保健机构标准化建设，建成8个危重孕产妇救治中心、8个新生儿救治中心。抓好国家医养结合试点市建设，建立居家养老示范区，新增养老床位4000张以上。完善食品质量追溯和安全保障体系，建立覆盖全市的标准化、规范化基层快检室，坚决把好饮食用药安全关口。推动全民阅读，建设“书香烟台”。大力弘扬社会主义核心价值观，实施“四德”工程和文明提升工程，争创全国文明城市“五连冠”。创新公共文化服务方式，繁荣文艺创作，建立文艺“双演”机制，传承和弘扬优秀传统文化，建设一批文化产业集聚区、知名文化企业和品牌。

（十三）推进社会治理创新，巩固和谐稳定局面。深入开展“三城联创”活动，争创全国社会治安综合治理优秀城市“七连冠”。深化“法德共进”，实施“七五”普法，完善公共法律服务体系。建设公共信用信息平台。充分发挥工会、共青团、妇联等群团组织在社会治理中的重要作用，做好信访、仲裁工作，健全社会矛盾排查预警和调处化解综合机制。强化公安基层基础建设，以信息化为支撑升级立体化社会治安防控体系，加大“天网工程”建设力度，加强反恐怖、反邪教和禁毒工作，严厉打击涉黑涉恶、盗抢骗和涉众型经济犯罪。加强基层政权和群众自治组织建设，培养社区工作者，组织好村（居）委会换届选举工作。提高应急管理水平，做好气象服务、防震减灾、防汛抗旱、森林防火及海上搜救等工作。全面落实安全生产企业主体责任、属地管理责任、部门监管责任，坚决遏制较大以上事故发生。落实援藏援疆和扶贫协作工作。加快市县档案馆建设，做好史志、民族宗教、红十字等工作。加强国防教育、国防动员、民兵预备役、人民防空和双拥共建工作，推动军民融合深度发展，巩固军政军民团结局面。

四、建设担当有为的人民政府

为人民服务，对人民负责，受人民监督，让人民满意，是人民政府必须始终坚守的信念。新一届政府要保持“赶考”姿态，坚定“考好”决心，交上满意答卷。

（一）坚持狠抓落实在状态。政府系统要在思想上敢于担当、在行动上注重实干、在方法上善于攻坚，集中精力抓重点、抓难点、抓突破。严格执行工作责任制，制定《推进工作落实实施方案》，建立职能性工作、突破性工作和战略性工作“三张任务清单”，使各项工作具体到单位、实化到项目、量化到指标，建立“事有专管之人、人有明确之责、责有限定之期”的工作体系。健全完善考核制度，严督查、严考核，真问责、真奖惩，坚决消除争权推责、扯皮掣肘等现象，坚决向庸懒散、不作为、慢作为等问题亮剑，让“想干事、敢干事、干成事”的人有舞台、上奖台，进一步浓厚攻坚克难抓落实、雷厉风行抓执行的干事创业氛围。

（二）坚持优化环境强服务。各级政府都要忠诚于党和人民的事业，所有政府部门的工作都必须服从大局需要、服从企业发展需要、服从群众和基层需要，再造工作流程，创新服务方式；所有政府工作人员都必须强化公仆意识，向前一步主动服务。深化“放管服”改革，继续削减和下放行政许可等权力事项，加强事中事后监管，推行“双随机一公开”，推动政务服务事项办理由实体政务大厅向网上办事大厅延伸。公开市县乡公共服务清单，开展“减证便民”专项行动，让企业、群众办事更方便、更省心。

（三）坚持依法行政严规范。认真执行人大决议决定，自觉接受人大法律监督和政协民主监督，主动接受社会监督和舆论监督。切实做好人大代表建议和政协提案办理工作。严格落实重大行政决策法定程序，认真听取法律顾问和社会各界意见建议。提高政府立法水平，完成安全生产监督管理、城市供水、地下空间建设用地管理等地方立法项目。深化行政执法监督体制改革，切实加强行政应诉工作。规范公共资源交易平台，推进交易过程信息化。严控“三公”经费和一般性支出，使每一笔财政资金都能花在明处、用出实效。提高政务公开制度化标准化水平，主动加强政策解读，及时回应社会关切。

（四）坚持从严治政守纪律。强化“四个意识”，严格履行全面从严治党政治责任，加强和规范党内政治生活，落实党内监督各项要求。坚持“一岗双责”，持续推进党风廉政建设和反腐败工作，把握运用好监督执纪“四种形态”，突出工程招投标、政府投融资、国有土地出让等重点领域和扶贫、低保、医保等民生领域，加强行政监察、审计监督和财政监管，筑牢制度“篱笆”，防范权力“越线”，努力建设廉洁政府。

各位代表，实干成就梦想，奋斗赢得未来。让我们更加紧密地团结在以习近平同志为核心的党中央周围，在中共烟台市委的坚强领导下，聚心惠民、聚力兴企、聚焦强市，为烟台率先发展、走在前列、决胜小康而努力奋斗，以优异成绩迎接党的十九大和省第十一次党代会胜利召开！
